# Supplementary figures and images for: Dysregulated lactate metabolism synergizes with ALS genetic risk factors to accelerate motor decline
Source: PLoS One. 2026 Apr 17;21(4):e0347135. doi: 10.1371/journal.pone.0347135 (PMC13089715; doi:10.1371/journal.pone.0347135)

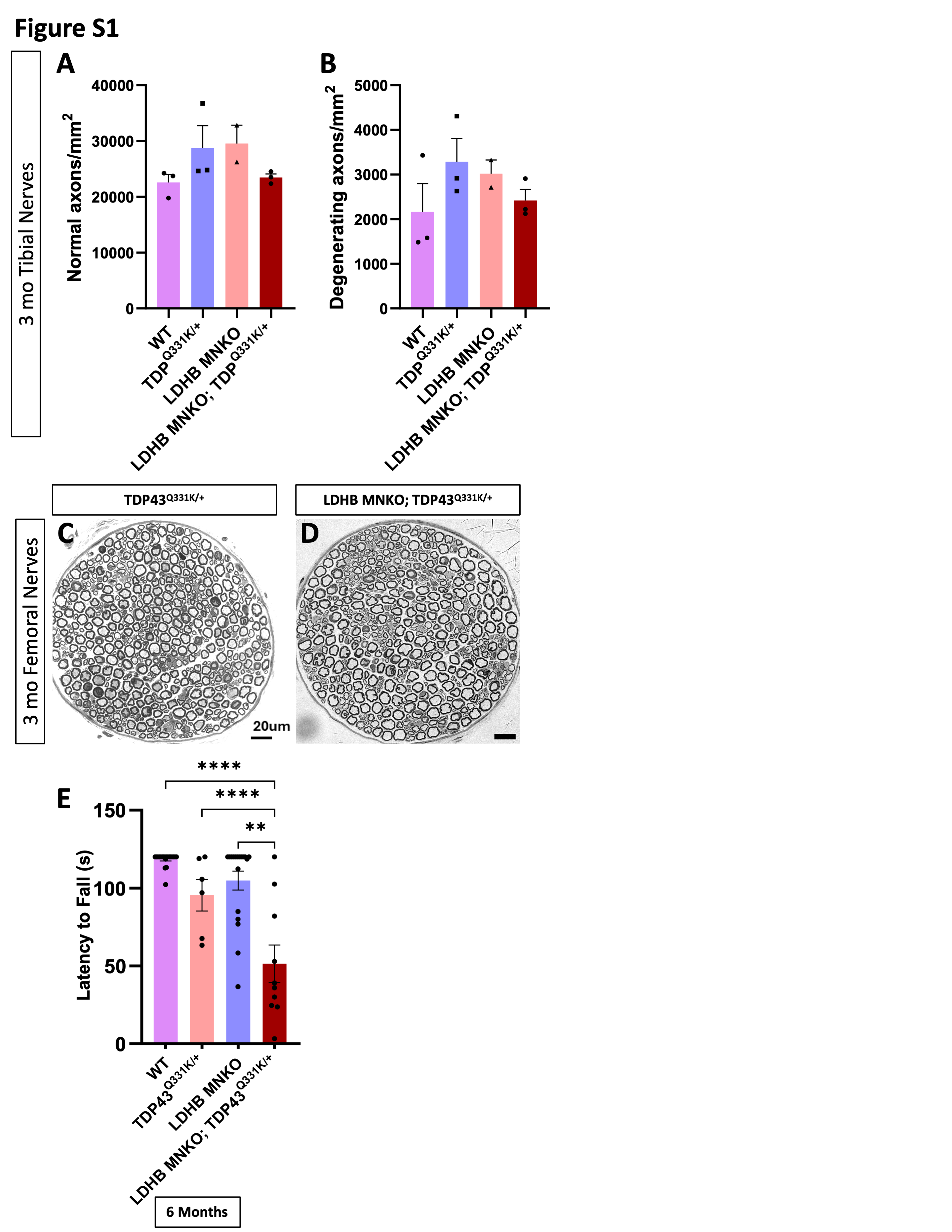

Supplement: S1 Fig — Representative images of 63x toluidine blue-stained sections of Femoral nerves from 3-month-old TDP43Q331K/+ and LDHB MNKO; TDP43Q331K/+ mice (C & D). Latency to fall from an inverted screen in 6-month-old animals (E). Statistical significance was determined by one-way ANOVA with Tukey’s multiple comparisons test, ***p < 0.001, **p < 0.01. (TIFF) [file pone.0347135.s001.tiff]

Original raw images  
Anti-flag Western blots  
Figure 3B

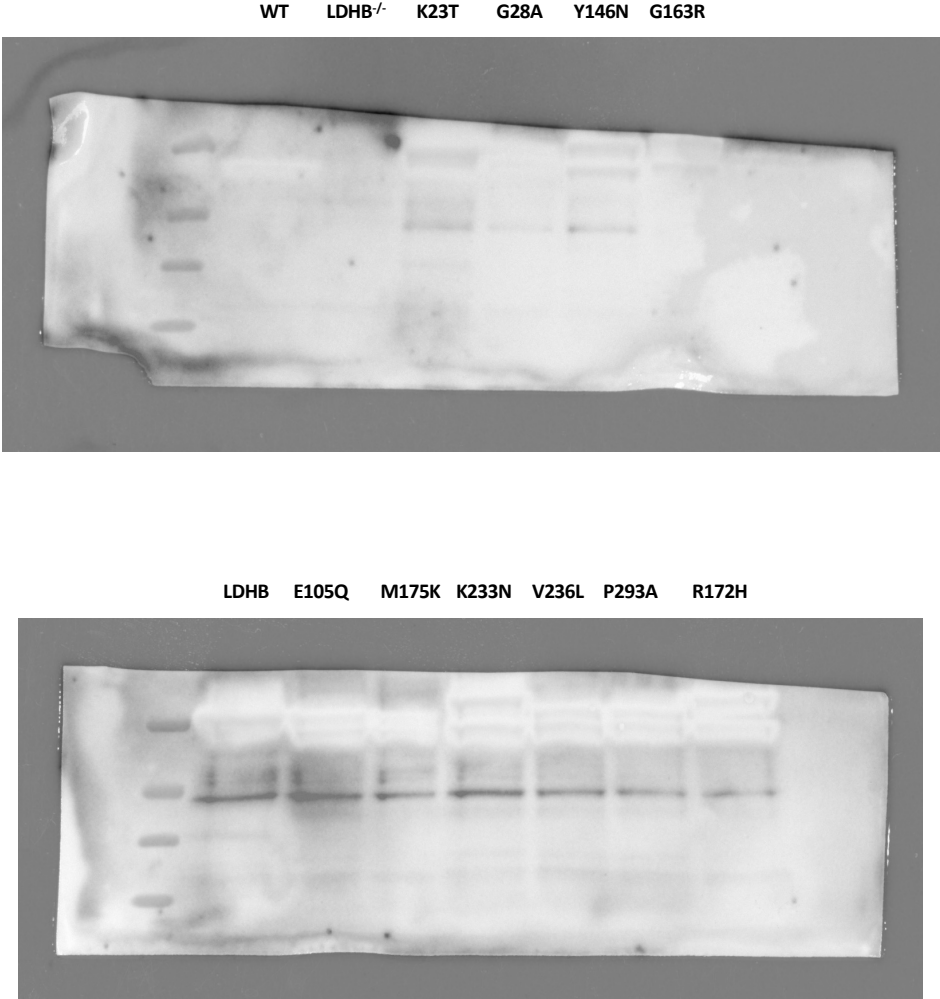

Supplement: S1 Raw Images — (PDF) [file pone.0347135.s003.pdf]
